# Identifying Gaps for Service Provision in Children and Young People With Learning Disability and Challenging Behaviours And/or Mental Health Needs

**DOI:** 10.1192/bjo.2022.420

**Published:** 2022-06-20

**Authors:** Nu Nu Yi, Joanne Watkins, Colin Welch, Zak Chowdhury, Sam Illaiee

**Affiliations:** North East London NHS Foundation Trust, London, United Kingdom

## Abstract

**Aims:**

Children and adolescents with learning disability need multi-disciplinary input when they present with challenging behaviours or mental health disorders. This patient group needs specialist skills from the clinicians and professionals to support and meet their needs. There is no commissioned Child and Adolescent Learning Disability Mental Health Service (CAMHS) in Waltham Forest and support comes from Specialist Tier 3 generic CAMHS which comprises of Emotional Difficulties Pathway, Neurodevelopmental Disorders Pathway and recently developed Behaviour Pathway which mostly comprises of specialist parenting training/interventions. To identify gaps in service provision for children and young people with learning disability presenting with challenging behaviours and/or mental health needs in Waltham Forest as there is no formally funded CAMHS learning disability service in the locality.

**Methods:**

All children and young people under 18 with learning disability under Waltham Forest CAMHS with ASD/ADHD and other neurodevelopmental disorders who meet the project criteria are included. Project criteria include 1) Main diagnosis of Learning Disability (Including clients with Learning difficulty (global), likely to have low IQ with cognitive impairment.), with or without associated other Neurodevelopmental disorders (e.g., ASD, ADHD or tics) or other mental health disorders and 2) Engages in challenging behaviours. Challenging behaviours defined as behaviours significantly limiting engagement in daily & family life, education and/or social activities, and have persisted for at least a period of 3 months. Data were collected from electronic recoding system of individual patients; using a data collection sheet on level of learning disability; comorbid neurodevelopmental or emotional and mental health disorders; profession of allocated clinician; joint working with discipline; involvement with social care; allied health professionals input; presenting difficulties and severity; CETR or hospital admissions; referral to National services; What interventions offered (Medications, Behaviour assessment and/or interventions); if medications offered, was it used as first line and how long for; parents’ view on medication management.

**Results:**

As we have expected, medication management were used as first line and there were limited offers of behaviour support. Joint working with social care, speech therapy and occupational therapy but with limited input especially occupational therapy in cases with high sensory needs. It was unclear with the cognitive assessment and diagnosing the learning disability in the population under 16s.

**Conclusion:**

There is a service gap for CAMHS learning disability population and more joint working needed among relevant health professionals.

